# Application of wood ash leads to strong vertical gradients in soil pH changing prokaryotic community structure in forest top soil

**DOI:** 10.1038/s41598-020-80732-0

**Published:** 2021-01-12

**Authors:** Toke Bang-Andreasen, Mette Peltre, Lea Ellegaard-Jensen, Lars Hestbjerg Hansen, Morten Ingerslev, Regin Rønn, Carsten Suhr Jacobsen, Rasmus Kjøller

**Affiliations:** 1 Department of Environmental Science, Aarhus University, Roskilde, Denmark; 2 Department of Biology, University of Copenhagen, Copenhagen Ø, Denmark; 3 Department of Geosciences and Natural Resource Management, University of Copenhagen, Frederiksberg C, Denmark; 4 Department of Plant and Environmental Sciences, University of Copenhagen, Frederiksberg C, Denmark

**Keywords:** Microbiology, Microbial communities, Environmental microbiology, Environmental chemistry, Forestry, Microbial ecology

## Abstract

Wood ash is alkaline and contains base-cations. Application of wood ash to forests therefore counteracts soil acidification and recycle nutrients removed during harvest. Wood ash application to soil leads to strong vertical gradients in physicochemical parameters. Consequently, we designed an experimental system where small-scale vertical changes in soil properties and prokaryotic community structure could be followed after wood ash application. A mixed fly and bottom ash was applied in dosages of 3 and 9 t ha^−1^ to the surface of soil mesocosms, simulating a typical coniferous podzol. Soil pH, exchangeable cations and 16S prokaryotic community was subsequently assessed at small depth intervals to 5 cm depth at regular intervals for one year. Wood ash significantly changed the prokaryotic community in the top of the soil column. Also, the largest increases in pH and concentrations of exchangeable cations was found here. The relative abundance of prokaryotic groups directionally changed, suggesting that wood ash favors copiotrophic prokaryotes at the expense of oligotrophic and acidophilic taxa. The effect of wood ash were negligible both in terms of pH- and biological changes in lower soil layers. Consequently, by micro-vertical profiling we showed that wood ash causes a steep gradient of abiotic factors driving biotic changes but only in the top-most soil layers.

## Introduction

The urge to limit the use of fossil fuels has led to an increased use of biomass, especially wood, for heat and energy production^1^; hence, increased application of wood ash in plantations may improve the sustainability of using biomass in heating- and power plants^1^. Wood ash can when applied to soil, counteract soil acidification and return valuable plant nutrients removed by harvest^2–4^. This is especially important in intensively managed silvicultural systems where continuous removal of wood for biofuel is particularly likely to cause soil acidification and nutrient loss^5^. Hence, increased application of wood ash in plantations improve the sustainability of using biomass in heating- and power plants. The relatively high concentration of harmful heavy metals (e.g. Cd and Pb) and the initial high alkalinity of wood ash may however cause adverse ecosystem effects that outweigh positive effects^4,6,7^. Soil prokaryotes contribute to key ecosystem processes involved in carbon and nutrient cycling and they are vital for plants and higher trophic levels of the terrestrial food web^8–11^. Changes in prokaryotic communities can therefore alter ecosystem processes^12–14^.

The effects of wood ash application on soil prokaryotes include changes in community composition, activity and quantity. These responses have generally been explained by an ash-induced raise in soil pH, increased concentrations of soil nutrients and possibly heavy metals, increased N-mineralization rates, and a change in amount or quality of dissolved organic carbon^6,15–23^. Soil pH is particularly important as a driver for prokaryotic community composition^24,25^. Changes in pH affect prokaryotes directly by changes in the osmotic conditions for the prokaryotic cells and indirectly by changing the pH-dependent solubility, and thus bioavailability, of mineral nutrients and toxic compounds^26,27^. Nutrient availability is also a major driver of prokaryotic community composition and taxonomic groups possessing copiotrophic lifestyles prosper upon increased nutrient availability at the expense of oligotrophic groups^28–31^.

Wood ash is typically applied on the soil surface leading to strong physicochemical gradients down the soil profile^32,33^. The vertical gradients in environmental parameters strongly affect microbial abundance and activity, and biochemical processes mediated by microorganisms^18,34–36^. Plants are also affected by this environmental variability as many roots selectively grow where nutrients are plentiful^37,38^. Thus, ash-induced changes in vertical gradients of environmental variables and nutrient availability can potentially affect biological processes, ecosystem structure and soil ecosystem services. Therefore, it is important to investigate the interaction between wood ash induced physicochemical gradients in top soils and the derived biotic consequences at the micro-vertical scale. Such investigations are presently scarce because assessment of wood ash effects on ecosystems typically relies on bulk sampling that combines soil from the upper 5–15 cm of the soil column, which may mask or average stratified effects^39,40^.

We therefore designed an experimental system to study small-scale vertical changes in soil properties and prokaryotic community structure after wood ash amendment. The experimental units were created by repacking homogenized material from the O- and A-horizon in two layers in large containers. This system allowed us to simulate the natural forest soil column in an otherwise spatially homogenous system optimized for repeated samplings over an entire year^33^. We hypothesized that the prokaryotic community composition would respond closely to wood ash induced pH and base cation gradients established down the soil profile. In particular, we expected that increased alkalinity in the uppermost soil layers would lead to lower abundance of acid-tolerant bacteria while favoring copiotrophic and alkaline-tolerant bacteria.

## Results

### pH and soil nutrients

The results regarding pH and soil nutrients are described in detail in Hansen et al^33^. and are briefly summarized here. Wood ash application significantly increased soil pH in the uppermost soil layers down to 2 cm depth (Table 1). One day after wood ash application the average soil pH at 0.5 cm depth was 3.26 ± 0.02 (SE), 5.79 ± 0.26and 10.02 ± 0.36in soils applied with 0, 3 and 9 t ha^−1^, respectively. Maximum pH values were found in these uppermost soil layers throughout the 1 year experimental period. Over time, the maximum pH decreased by approximately pH 0.006 day^−1^ in both the 3 and 9 t ha^−1^ amended soil. No significant pH changes occurred at lower soil depths and no changes were observed in unamended soil.

**Table 1:** Average pH values in the different soil depths corresponding to the soil samples used for sequencing of the prokaryotic community across wood ash concentrations and time points (mean ± SE).

	0 t ha-1					3 t ha-1					9 t ha-1				
	1 d	62 d	152 d	278 d	363 d	1 d	62 d	152 d	278 d	363 d	1 d	62 d	152 d	278 d	363 d
0.5 cm	3.26 ± 0.02	3.50 ± 0.11	3.46 ± 0.11	3.31 ± 0.05	3.59 ± 0.10	5.79 ± 0.26	7.37 ± 0.19	7.81 ± 0.16	6.26 ± 0.12	5.05 ± 0.06	10.02 ± 0.36	8.09 ± 0.12	7.75 ± 0.10	7.44 ± 0.09	7.65 ± 0.07
1.5 cm	3.10 ± 0.01	3.26 ± 0.00	3.24 ± 0.00	3.35 ± 0.01	3.55 ± 0.01	3.27 ± 0.06	4.20 ± 0.09	4.44 ± 0.14	4.44 ± 0.05	4.28 ± 0.03	5.19 ± 0.16	4.92 ± 0.20	5.22 ± 0.17	5.38 ± 0.11	5.84 ± 0.19
2.5 cm	3.00 ± 0.00*	3.25 ± 0.00	3.28 ± 0.00	3.40 ± 0.00	3.57 ± 0.00	3.17 ± 0.02	3.63 ± 0.01	3.85 ± 0.01	4.00 ± 0.02	4.01 ± 0.01	3.54 ± 0.08	3.86 ± 0.02	4.01 ± 0.01	4.35 ± 0.03	4.58 ± 0.02
4.5 cm	3.01 ± 0.00	3.23 ± 0.01	3.34 ± 0.00	3.44 ± 0.01	3.56 ± 0.00	3.08 ± 0.00	3.46 ± 0.00	3.72 ± 0.00	3.66 ± 0.01	3.76 ± 0.00	2.79 ± 0.00	3.56 ± 0.01	3.82 ± 0.01	3.89 ± 0.01	4.11 ± 0.01

The soil pH was measured using 500 µm steps down the soil profile (see “Methods” section) and the presented values are calculated as the average pH measured in the depth intervals of [0–0.95 cm cm], [1–1.95 cm], [2–2.95 cm] and [4–4.95 cm] each consisting of 20 pH measurements. The full picture of the measured pH across all depths and time points (monthly intervals) are found in Hansen et al^33^.

*< 0.01.

Cation exchange capacity (CEC) increased significantly in the upper 1 cm of the soil column of the 3 t ha^−1^ ash amended soil and in the upper 2 cm of the 9 t ha^−1^ ash amended soil when compared to the unamended soil (Supplementary Table 1). Concentrations of exchangeable cations (Ca, K, Mg and Mn) increased within the top 0–5 cm of the 9 t ha^−1^ soil column and in the top 0–1 cm of the 3 t ha^−1^ soil column. The same was true for exchangeable Na with the exception that Na did not show any increase in 1–2 cm depth of soil applied with 9 t ha^−1^. In contrast to the other cations, exchangeable Fe and Al generally decreased in wood ash amended soils (Supplementary Table 1).

### Prokaryotic community

A total of 3.7 million DNA sequences remained after bioinformatic processing. When separated into 170 samples the sequencing depth ranged from 5071 to 74,746 sequences per sample. A total of 11,828 unique Operational Taxonomic Units (OTUs) were derived from these sequences based on the 97% clustering criteria.

**Figure 1:**
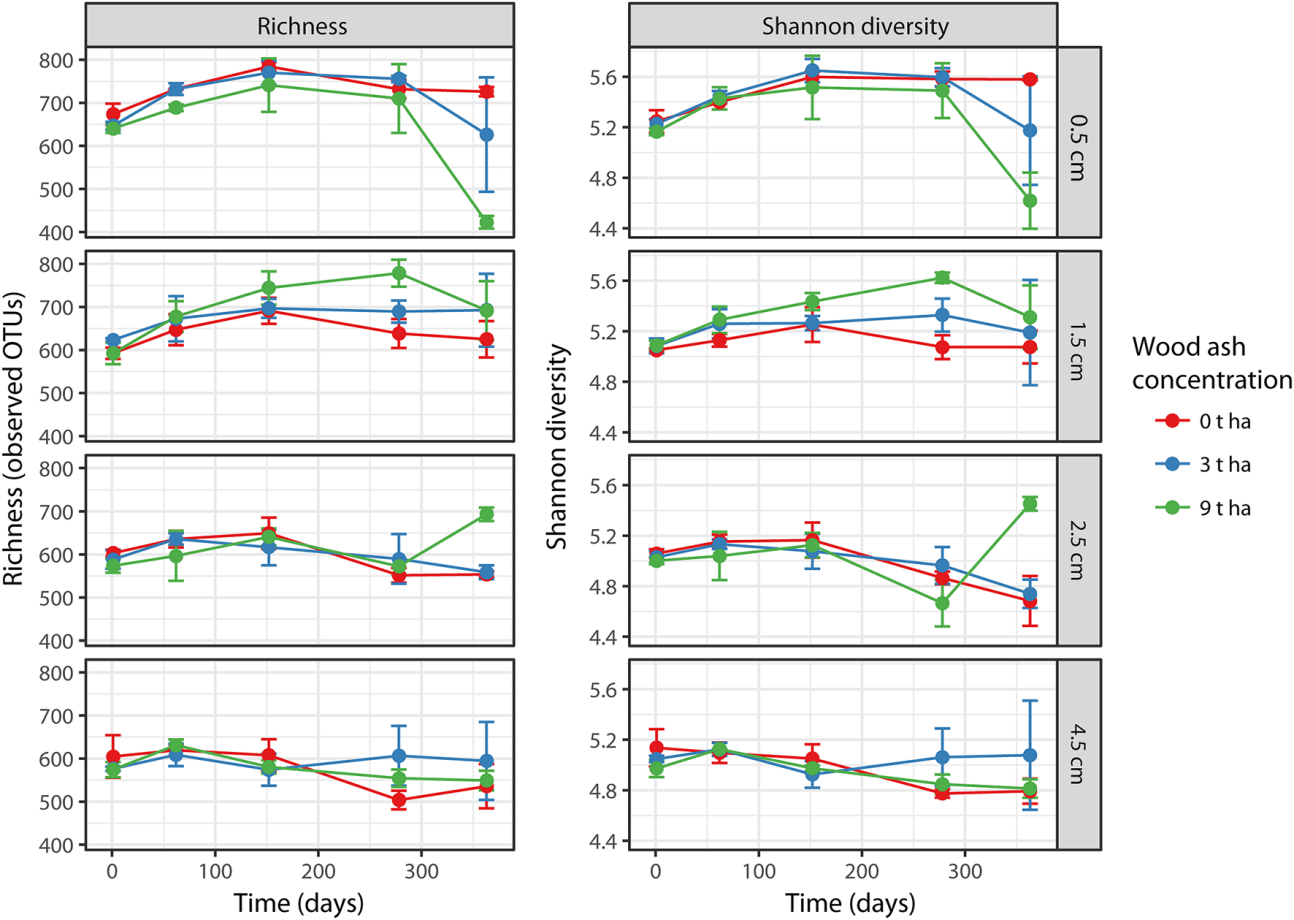
Prokaryotic richness (observed OTUs) and Shannon diversity across wood ash concentrations, soil depth and time after wood ash application. Error bars represent standard errors (n = 2 or 3). Figure was created in R v. 1.0.153 (www.R-project.org) and GIMP v. 2.8.22 (https://www.gimp.org).

Overall, prokaryotic richness (number of observed OTUs) and Shannon diversity significantly (*p* < 0.001) decreased with soil depth across all sampling-times and wood ash doses (Fig. 1). These metrics did not significantly change in the unamended soil throughout the experimental period. In comparison to the unamended soil the highest dose of ash application (9 t ha^−1^) significantly (*p* < 0.05) decreased richness and Shannon diversity at 0.5 cm soil depth after 363 days. In contrast, Shannon diversity significantly (*p* < 0.05) increased at 2.5 cm soil depth 363 days after 9 t ha^−1^ ash application when compared to the unamended soil. The 3 t ha^−1^ ash amendment did not result in significant differences in these metrics when compared to the unamended soil.

**Figure 2:**
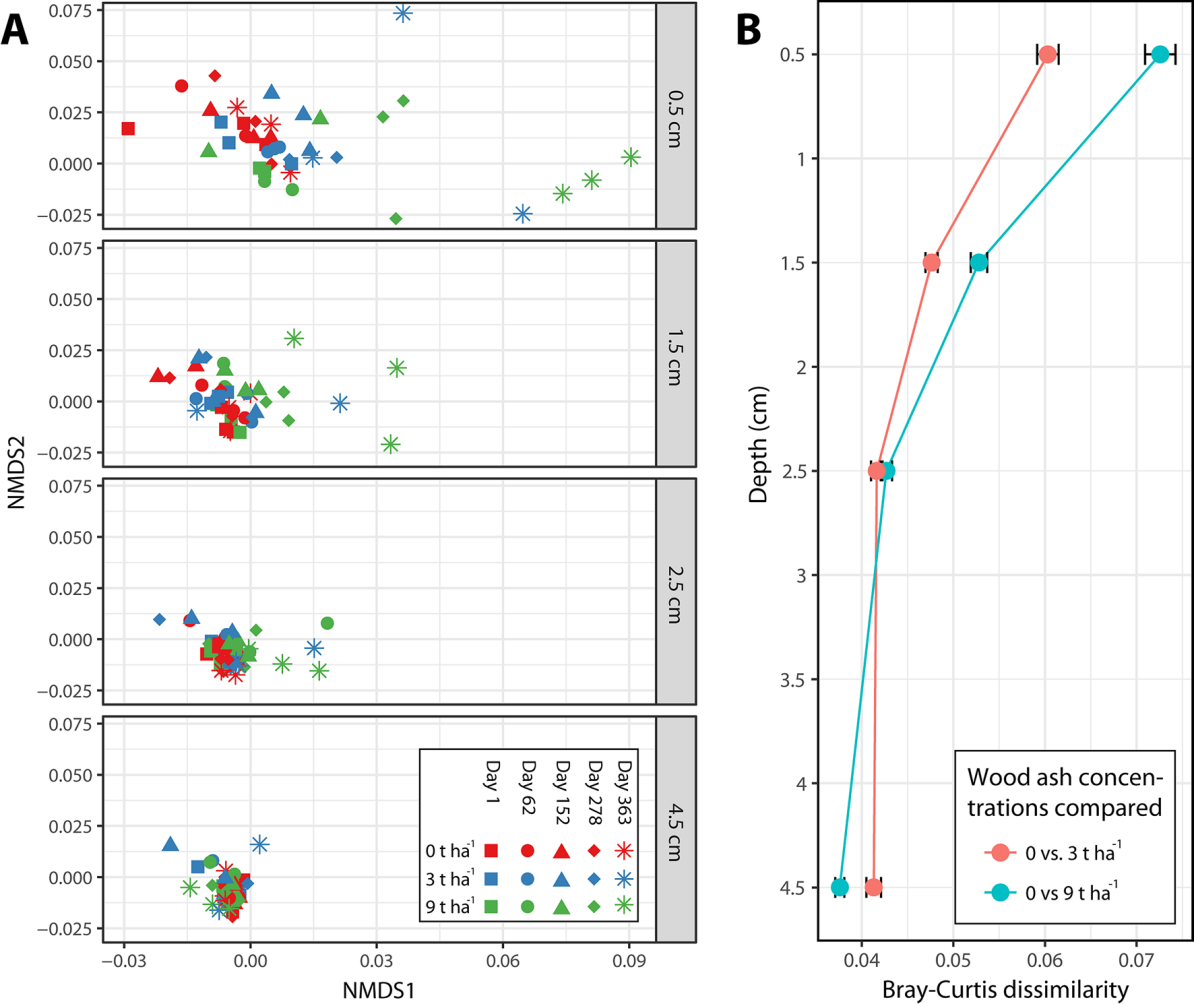
Bray–Curtis dissimilarity of prokaryotic communities across wood ash concentrations, time after ash application and depth below soil surface. (**A**) NMDS projection of dissimilarities between samples separated into depths (stress = 0.11). (**B**) Average Bray–Curtis dissimilarity between prokaryotic communities of unamended soil samples compared to samples applied with ash doses of 3 and 9 t ha^−1^, respectively (0 vs. 3 t ha^−1^ and 0 vs. 9 t ha^−1^), within each soil depth. Figure was created in R v. 1.0.153 (www.R-project.org) and GIMP v. 2.8.22 (https://www.gimp.org).

The prokaryotic community composition changed in response to wood ash application. The most pronounced effects occurred in the uppermost soil layer and the effect increased with time (Fig. 2A,B; Supplementary Table 2).

At the beginning of the experiment the prokaryotic community was dominated by Acidobacteria (77.6% relative abundance across all soil depths), Proteobacteria (8.2%), candidate division WPS-2 (5.6%) and Verrucomicrobia (3.2%) (Supplementary Fig. 1).

**Figure 3:**
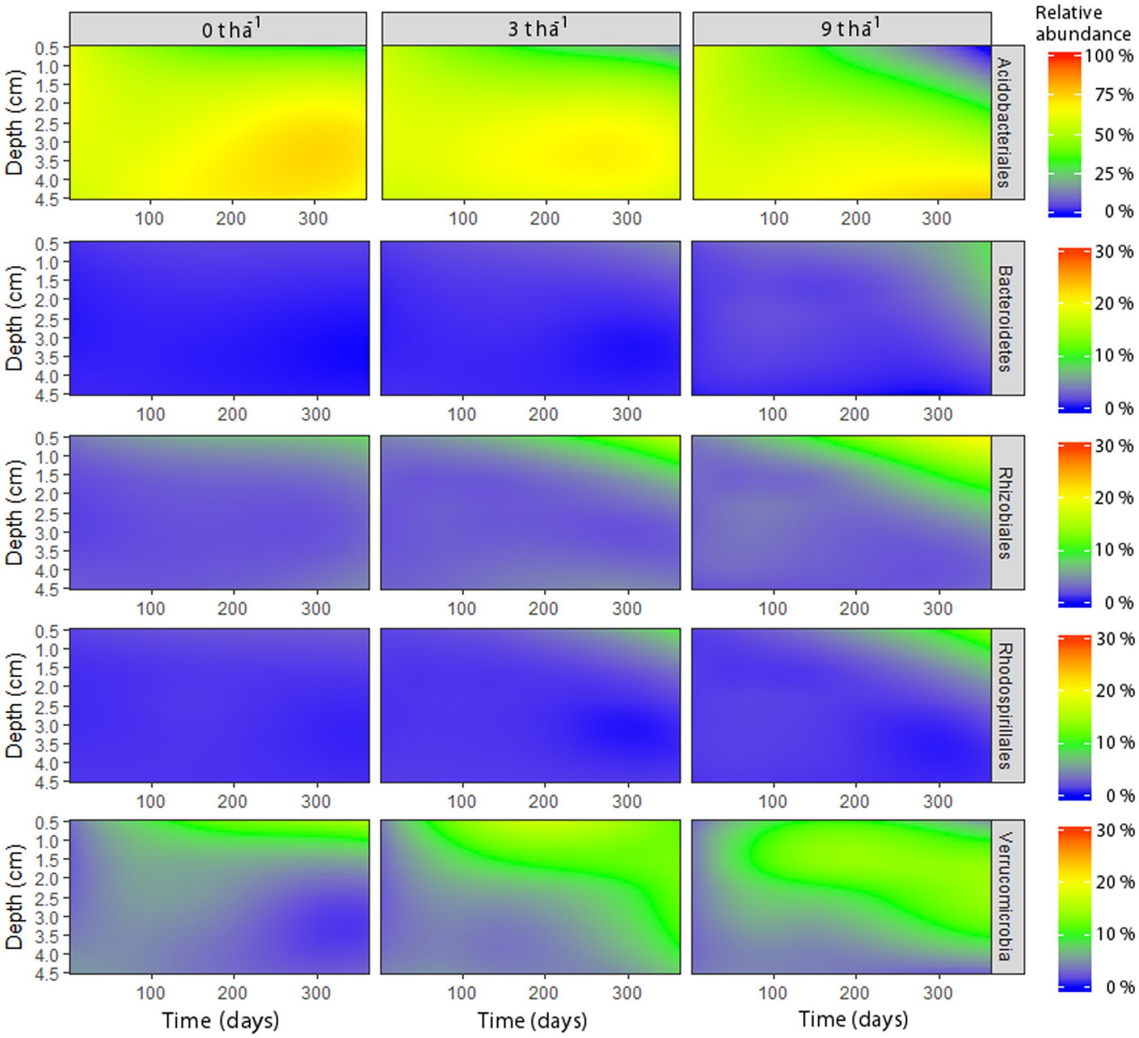
Contour plots of the relative abundance of dominant prokaryotic groups across wood ash concentration, soil depth and time after wood ash application. Colors refer to relative abundance of the groups. Note different color scales between Acidobacteriales and the other presented taxa. Figure was created in R v. 1.0.153 (www.R-project.org) and GIMP v. 2.8.22 (https://www.gimp.org).

Wood ash application resulted in a significant and trending decrease in relative abundance of Acidobacteria at soil depth 0.5 cm (*p* = 0.04) and 1.5 cm (*p* = 0.08) respectively, while no responses were observed at lower soil layers (Supplementary Fig. 1). Acidobacteriales contributed the most to this decrease (Fig. 3). Other groups increased in relative abundance in the uppermost soil layers in response to wood ash application: Alphaproteobacteria (*p* = 0.04 and *p* = 0.09 at 0.5 and 1.5 cm depth, respectively), Deltaproteobacteria (*p* = 0.04 at 0.5 cm), Verrucomicrobia (*p* = 0.03 and *p* = 0.07 at 0.5 and 1.5 cm depth, respectively) and Bacteroidetes (*p* = 0.08 at 1.5 cm depth). The most pronounced increase was seen for Alphaproteobacteria with the orders Rhizobiales and Rhodospirillales contributing most to this increase (Fig. 3). No significant or trending changes in relative abundance occurred for these dominant groups in deeper soil layers (p > 0.1).

### Physicochemical parameters and community responses

The strongest correlations of measured physicochemical parameters to the NMDS projections of community dissimilarities (*Bioenv* analysis) occurred in the uppermost soil (0.5 cm) (Table 2). Similarly, the highest number of physicochemical parameters significantly explaining community dissimilarities between samples (*Adonis* analysis) was observed in the uppermost soil. Thus, at a soil depth of 0.5 cm, pH, CEC and exchangeable Al, Ca, Fe, Mg and Mn explained the observed community dissimilarities between samples (Table 2). At 1.5 cm soil depth, only pH, CEC and exchangeable Ca, Mg and Mn were selected as significant parameters, while only Ca was important at 2.5 and 4.5 cm soil depth.

**Table 2:** R2-values of Envfit and Adonis (PERMANOVA) analyses.

Explanatory variable	0.5 cm		1.5 cm		2.5 cm		4.5 cm	
	*Envfit*	*Adonis*	*Envfit*	*Adonis*	*Envfit*	*Adonis*	*Envfit*	*Adonis*
pH	0.39**	0.19***	0.42*	0.13**	0.02	ns	0.09	ns
CEC	0.19*	ns	0.26*	ns	0.11	ns	0.07	ns
Al	0.39**	ns	0.13	ns	0.00	ns	0.03	ns
Ca	0.37**	0.05*	0.30*	ns	0.10	0.10**	0.11	0.02*
Fe	0.30*	ns	0.06	ns	0.07	ns	0.22	ns
K	0.14	ns	0.06	ns	0.05	ns	0.02	ns
Mg	0.19*	0.15***	0.25*	ns	0.03	ns	0.05	ns
Mn	0.39**	ns	0.22*	ns	0.03	ns	0.05	ns
Na	0.14	ns	0.01	ns	0.04	ns	0.02	ns

The Envfit analysis test for significant correlation of environmental parameters to the NMDS projections of community dissimilarities between samples (Bray–Curtis) each parameter separately. The Adonis analysis test whether environmental parameters significantly explain the observed community dissimilarities (Bray–Curtis) between samples and uses a statistical model that includes all environmental parameters. Values are R2-values.

Asterisks refers to significance level (* is 0.01 < *p* < 0.05, ** is 0.001 < *p* < 0.01, *** is *p* < 0.001). “ns” are non-significant results from the Adonis test.

## Discussion

Soil pH is a main driver of microbial community structure^13,25,41,42^ and we hypothesized that prokaryotic richness and community composition would closely follow an expected strong wood ash induced pH gradient. We did indeed record a strong vertical pH gradient ranging from pH 4.5 – 11 within the top 1–1.5 cm of the soil and this drove a likewise marked change in the prokaryotic community structure. Previous studies have demonstrated that the high alkalinity of wood ash is one of the main reasons for wood ash induced changes in soil biota^6,15,17,23^. Similarly to the present study, Gömöryová et al^32^. found a strong gradient of physicochemical changes in the top soil column after wood ash application coupled with increased heterogeneity of the culturable microbial functional community structure assessed by BIOLOG plates. Combined, the results of Gömöryová et al^32^. and the present study emphasize that strong and highly stratified biotic and abiotic responses occur at the mm-cm scales down the top soil after wood ash application. Changes in amount and quality of organic matter and the biological and physical mixing with the mineral soil below are known to affect the composition of soil biota and the biochemical processes they mediate^18,43–45^. For instance, microbial biomass is greatest in surface layers^34–36^ and stimulation of copiotrophic microbes in these top layers may have profound effects on the decomposition rate.

In general, microorganisms are adapted to the prevailing pH of their environment^46^ and the pH increase in the top soil of our experiment caused extreme environmental conditions for most of the indigenous microbial community. The extreme changes in pH and concentration of exchangeable cations lead to drastic changes in osmotic conditions that are probably harmful for many prokaryotic cells in the soil. Most prokaryotes have a growth range within 3–4 pH units and pH levels outside this range are detrimental^47,48^. This is exemplified by Acidobacteria which dominate in low pH soils and are negatively affected by increasing pH^13,25,49^. The decrease in Acidobacteria was accompanied by an increase in the relative abundance of Bacteroidetes and Alphaproteobacteria. These groups have previously been found to respond positively to increasing pH in soils^25,42,50^. Indirect effects of the increased pH include changed solubility (and thus bioavailability) of nutrients and toxic compounds (e.g. heavy metals) due to the pH-dependent equilibria of these compounds in the soil matrix^27,51,52^. These indirect pH effects probably also contribute to the observed prokaryotic changes.

Increased levels of bioavailable nutrients, here measured as exchangeable cations, and increasing CEC in the top soil layers after ash application, probably also affected the prokaryotic communities. This is indicated by the stimulation of taxonomic groups generally known to possess copiotrophic lifestyles including Alphaproteobacteria, and Bacteroidetes^28,53–55^. Within Alphaproteobacteria, especially the order Rhizobiales increased in relative abundance after wood ash application. Many members of Rhizobiales are copiotrophs^56,57^ and able to cope with high levels of heavy metals^58^. These capabilities probably give members of Rhizobiales advantages over other microorganisms upon the addition of ash-inherent nutrients and heavy metals. Wood ash amendment can also indirectly increase bioavailable carbon and nitrogen sources, which probably also contribute to the observed stimulation of copiotrophic groups^23,26,52^. The relative increase in copiotrophic groups are probably also enhanced by the relative decrease in the oligotrophic Acidobacteriales as the copiotrophic groups will outcompete the slower growing oligotrophic groups when bioavailable nutrients are plentiful.

While most of the prokaryotic community responses occurred 0.5–1.5 cm below the soil surface, the phylum Verrucomicrobia also increased in relative abundance deeper in the soil profile. Members of Verrucomicrobia generally possess oligotrophic lifestyles^31^, but the vast majority of this phylum remains undescribed leaving their functional roles in soil largely unknown^59^. However, wood ash addition also affected the soil environment in deeper soil layers (2.5 and 4.5 cm) as seen by the significant increases of cations at 9 t ha^−1^ ash application. This may partly explain the increase in relative abundance of Verrucomicrobia but not why this should favor this bacterial group over other groups (who remain largely unchanged at these soil depths). More investigations are needed to reveal the possible interaction of Verrucomicrobia and other wood ash induced changes in physicochemical parameters.

Temporal responses were evident after wood ash application with the communities separating more and more from the initial assembly with time. This was also true for prokaryotic diversity, as the only significant effects of wood ash application on diversity were observed one year after ash application. In contrast, the most pronounced effects on pH and cations were observed one day after wood ash application after which pH and cation concentrations gradually decreased with time. These temporal responses suggest that parts of the prokaryotic community slowly adapts to the new environmental conditions created by the wood ash application. We can only speculate if this separation with time would continue after one year, but as wood ash application produces year-long alterations of the top-soil chemistry^60,61^ this seems likely.

As discussed above, copiotrophic groups likely proliferate and utilize the increased concentration of available nutrients at the expense of oligotrophic groups. However, the dramatic environmental changes in the top layers of the soil observed here would suggest a more rapid prokaryotic response as for example the high pH should cause cell lysis of many prokaryotes. A possible explanation for the relatively slow community responses observed is the inclusion of relic DNA from dead prokaryotes. Extracellular DNA is relatively stable and only slowly degrades in soils^62,63^ and the inclusion of DNA from dead organisms will thus indicate slower community responses than what is actually true in the soil^64^. Total RNA-sequencing would circumvent the bias associated with relic DNA as RNA have much faster turnover time^65–67^. We therefore suggest that future studies should also consider RNA-based techniques when studying short-time reactions. An additional advantage of direct- as opposed to PCR-based sequencing is the inclusion of potential competitors (e.g. fungi) as well as grazers (e.g. protozoa) in the sequenced data pool.

The marked changes in abiotic and biotic parameters in the top soil after wood ash application potentially affect biochemical processes important for soil ecosystem functioning. Accordingly, Gömöryová et al^32^. showed a vertical stratification in the relative abundance of different microbial functional groups after wood ash application. These stratified responses need to be taken into consideration to identify environmental impacts of wood ash application. However, assessments of wood ash effects on soil ecosystems are typically performed on un-stratified samples of the top 5–15 cm of soil^16,21,22,44,45^. This sampling strategy strongly underestimate the marked responses observed just below soil surface. More studies are needed to reveal if the pronounced effects in the uppermost soil layers after wood ash application cause significant effects within the overall ecosystem, and hence, whether the use of un-stratified bulk sampling in assessment of wood ash application needs to be replaced by sampling of soil profiles in higher vertical resolution. Finally, we also note that in forestry the spreading of wood ash with heavy machinery will cause a much more inhomogeneous distribution pattern both on the surface but potentially also vertically via soil pores than what was observed in this study.

## Conclusion

Wood ash application creates strong vertical gradients in environmental parameters down the uppermost part of the soil profile. This is seen as large increases in pH and exchangeable cation concentrations just below the soil surface with less pronounced increases at deeper soil layers. Soil prokaryotic community composition is directly altered by this environmental gradient and shows dramatic community shifts just 0.5 and 1.5 cm below the soil surface. Community responses within these layers increase in magnitude throughout a period of one year following wood ash application. Prokaryotic groups known to thrive at higher pH levels and with copiotrophic lifestyles increased in dominance at the expense of acidophilic and oligotrophic prokaryotic groups. We therefore question the typically used un-stratified bulk sampling of soils in the assessment of ecosystem effects after ash application. Bulk sampling disregards the highly stratified biotic and abiotic responses after wood ash application. Future investigations should examine the strong vertical gradients of soil conditions created by wood ash application, and whether higher resolution profiling is needed to fully assess the effects of ash addition on soil ecosystems.

## Methods

### Soil collection and experimental set-up

Soil was collected from a Norway spruce (*Picea abies* (L.) Karst.) plantation, “Gedhus Plantage” (56°16′39″N, 09°05′10″E), in late August 2014. The plantation is a second generation forest on former heathland with a podsolized soil formed on a well-drained, sandy glacial till. The climate includes a mean annual precipitation of 850 mm and a mean annual temperature of 8.4 °C. Approximately 30 and 300 kg of soil from the O- and A-horizon, respectively, was sampled, placed into plastic containers and stored outside in dry and shady conditions before establishing the experimental system. Table 3 presents physicochemical composition of the soil horizons. In late November 2014 the soil was sieved (4 and 2 mm mesh for O- and A-horizon, respectively) and thereafter re-packed into nine boxes (42 × 72 cm) to simulate an intact forest soil. Soil was packed so the top 1-cm was organic O-horizon soil with density 0.2 g cm^3^ on top of 15 cm of mineral A-horizon soil with density 0.65 g cm^3^. The A-horizon soil contained > 90% sand, little clay and 6% organic matter^33^.

**Table 3:** Physicochemical composition of the applied wood ash and the used O- and A- horizon soil.

	Wood ash	O-Horizon	A-Horizon
pH	12.7	3.78	3.82
Total C (g kg^−1^)	66.4	397	50.1
Total N (g kg^−1^)	0.70	13.9	1.52
CEC (Cmol(+) kg^−1^)	–	12.9	4.48
Al (mg kg^−1^)	12.4	1.01	823
Fe (mg kg^−1^)	6.72	1.11	584
Ca (mg kg^−1^)	135	1.54	131
K (mg kg^−1^)	39.4	628	378
Mg (mg kg^−1^)	12.7	573	22.2
Mn (mg kg^−1^)	7.43	21.4	20.9
Na (mg kg^−1^)	10.5	310	85.7

All metal concentrations are total concentrations following acid digestions and subsequent ICP-MS. Presented values represents averages (n = 3). Data are extracted from Hansen et al^33^. and Maresca et al^27^.

Wood ash was evenly distributed on top of the soil in doses corresponding to 0, 3 and 9 t ha^−1^ (dry weight ash / dry weight soil). Current regulation in Denmark sets the maximal limit for wood ash application to 3 t ha^−1^ three times during a 70 year growth cycle, with a minimum time period between applications of 10 years. Triplicate mesocosms were established for each ash dose. The ash was a mixed bottom and fly ash from a heating plant in Brande, Denmark, fueled with coniferous wood chips. Table 3 presents physicochemical composition of the ash (for more extensive chemical composition, see ash “MA-9c” in^27^. For more details on the experimental design, see Hansen et al^33^.

Above each box we established a watering system consisting of 18 timer-regulated dripping nozzles that applied demineralized water equivalent to the yearly precipitation of the forest site divided in weekly irrigation events. Filter paper was placed on top of stainless steel grids that were positioned 5 cm above soil surface to ensure evenly distribution of water onto soil surface. Gravel stones under the soil allowed water drainage through a hole in the bottom of the boxes. The boxes were incubated in a laboratory at room temperature (≈ 20 °C) under aerobic conditions for an experimental period of 363 days.

### pH and CEC measurements

Once a month, soil cores were collected using plastic tubes (Ø = 6 cm). A microsensor (pH-500, Unisense, DK) measured pH down the center of the sampled cores at 500 µm steps to 5 cm depth. A second set of soil cores were retrieved after 62, 152, 278 and 363 days after ash application and separated into soil depths of 0–1, 1–2, 2–3, and 4–5 cm which were extracted with 1 M NH_4_NO_3_ to analyze for exchangeable base cations (Al, Ca, Fe, K, Mg, Mn, Na) (Thermo Scientific, iCAP Q, ICP-MS). Empty tubes were left in the holes to keep structure of soil intact throughout the study. For more details see Hansen et al^33^.

### Prokaryotic community: soil sampling, DNA extraction and library preparation

Soil samples (0.5 g) from 4 soil depths (0.5, 1.5, 2.5 and 4.5 cm) were collected for DNA extraction at four sampling times (1, 62, 152, 278 and 363 days) after wood ash application, for a total 180 samples.The soil samples were retrieved with a special procedure to avoid inclusion of microorganisms and ash that may potentially be dragged down into the soil when pushing a soil corer into the soil (Supplementary Fig. 2)^43^. Soil samples were immediately frozen in liquid nitrogen and stored at − 80 °C.

DNA was extracted using the PowerLyzer PowerSoil DNA Isolation Kit (Mobio, Carlsbad, CA, US) following manufacturer’s protocol. DNA concentrations were measured using the Qubit HS kit (Invitrogen, Carlsbad, CA, US) to ensure DNA yield of 1 to 10 ng/µl. DNA extracts were stored at -20 °C prior to 16S rRNA gene library preparation.

DNA extracts were prepared for 16S rRNA gene amplicon sequencing using a two-step amplification procedure. The first PCR run amplified the 16S rRNA gene using the primers 341f.: 5′-CCTAYGGGRBGCASCAG-3′ and 806r: 5′-GGACTACNNGGGTATCTAAT-3′ which flanks the V3-V4 region of the 16S rRNA gene 40. The forward and reverse primers contained Nextera overhang (Illumina Inc., CA, US) which allowed the addition of multiplexing indices in the second PCR. The first PCR used a master mix consisting of 12 µl Accuprime SuperMix II (Thermo), 0.5 µl of each primer (10 µM each), 2.0 µl bovine serum albumin (10 mg ml^−1^; Bioron, Ludwigshafen, Germany) and 5 µl DNA template. Amplification was carried out on a Bio-Rad CFX-96 (Bio-Rad, Hercules, CA, US) with thermal conditions of 95 °C for 2 min followed by 33 cycles of 95 °C for 15 s, 55 °C for 15 s, 68 °C for 40 s and ending with a final elongation of 68° for 4 min. Amplification of 16S rRNA gene products were confirmed by gel-electrophoresis. Second PCR added indexes i7 and i5 and adapters by using primers described in the Nextera XT indexing kit (Illumina) which targets the overhang described above. Mastermix used for the second PCR was 12 µl AccuPrime SuperMix II (Invitrogen), 2 µl of each index primer (unique combination per sample), 5 µl amplicon product from first PCR and 7 µl sterile water. Thermal conditions for the second PCR run was 98 °C for 1 min followed by 13 cycles of 98 °C for 10 s, 55 °C for 20 s, 68 °C for 40 s and a final elongation of 68 °C for 5 min. The resulting amplicons were then cleaned using HighPrep PCR reagent (MAGBIO Genomics. Gaithersburg, USA) following Illumina preparation instructions. The cleaned amplicons were visualized using gel-electrophoresis. All amplicons were then, in equimolar concentrations, pooled into the final 16S library, spiked with 10% PhiX, and sequenced on Illumina MiSeq using the V2 reagent kit (250 bp; paired end). Sequences is available at Sequence Read Archive under the project accession PRJNA541177.

### Processing of DNA sequences

Paired end sequences were merged with a minimum overlap of 20 bp using PEAR v0.9.8^68^. Removal of primers, cutting of bases from both ends of sequences with phred quality score below 25 and removal of sequences shorter than 380 bp was done using cutadapt v1.9 41. Further processing of sequences was carried out with the UPARSE pipeline 42 using default settings as set in USEARCH version 7.0.1090. Sequences were clustered into operational taxonomic units (OTUs) based on 97% sequence similarity threshold. Chimeras were removed denovo and reference based using UCHIME against the SILVA v123.1 16S database 43. OTUs were taxonomically classified using the RDP classifier 44 against the Greengenes reference database v13.8 45. Singleton OTUs were removed and 10 samples with less than 5000 sequences were discarded.

Richness (number of unique OTUs) and Shannon diversity were calculated based on a rarified OTU table (5000 sequence per sample) using the vegan package 46 in R 47. Differences in prokaryotic communities between samples were calculated using Bray–Curtis dissimilarity on a DeSeq2 normalized 48 OTU-table and eventually visualized using NMDS.

### Statistics

We used one-way ANOVA with repeated measurements to test for differences in soil pH, CEC and exchangeable cations in the different soil depths in each of the three ash treatments. Sphericity and normal distribution of data was tested using Mauchly’s and Shapiro–Wilk tests, respectively^33^.

We used three-way ANOVA to test for significant differences in richness and Shannon diversity using ash concentration, time and soil depths as independent variables. Input data for the two- and three-way ANOVAs were tested for homogeneity of variance and normal distribution using Levene’s and Shapiro–Wilk tests, respectively.

Within each soil depth, differential abundance of prokaryotic taxa were tested using Kruskal–Wallis analysis with the interaction between ash concentration and time as the explanatory parameter. This was done at taxonomic ranks from phylum to genus. Kruskal–Wallis test was applied because the OTU data was not normally distributed. Benjamini–Hochberg procedure was used for false discovery rate adjustments.

Correlations of soil pH and CEC to NMDS projections of community dissimilarities were performed with the R package vegan 46 using the function Envfit with Benjamini–Hochberg p-value adjustment. Furthermore, the explanatory strength of pH, CEC and exchangeable cations on the observed community dissimilarities were evaluated using the function Adonis, which performs permutational analysis of variance (PERMANOVA; 10,000 permutations) with Bray–Curtis dissimilarities as response variable. We used a forward selection strategy to ensure that only explanatory variables with significant p-values were included in the Adonis models. Envfit and Adonis tests were performed for each soil depth separately. Adonis testing was also used to assess whether applied wood ash doses and time after wood ash application significantly could explain the observed variation in Bray–Curtis dissimilarities between samples.

Statistical analyses were regarded significant and trending at *p* < 0.05 and 0.05 < *p* < 0.1, respectively.

## Supplementary Information

Supplementary Information.

## References

[1] Silva FC, Cruz NC, Tarelho LAC, Rodrigues SM (2019). Use of biomass ash-based materials as soil fertilisers: critical review of the existing regulatory framework. J. Clean Prod..

[2] Huotari N, Tillman-Sutela E, Moilanen M, Laiho R (2015). Recycling of ash—for the good of the environment?. Forest Ecol. Manag..

[3] Ingerslev M, Skov S, Sevel L, Pedersen LB (2011). Element budgets of forest biomass combustion and ash fertilisation—a Danish case-study. Biomass Bioenergy.

[4] Karltun, E. *et al.* in *Sustainable Use of Forest Biomass for Energy* (eds Röser, D., Asikainen, A., Raulund-Rasmussen, K. & Stupak, I.) 79–108 (Springer, Berlin, 2008).

[5] Thiffault E (2011). Effects of forest biomass harvesting on soil productivity in boreal and temperate forests—a review. Environ. Rev..

[6] Aronsson KA, Ekelund NGA (2004). Biological effects of wood ash application to forest and aquatic ecosystems. J. Environ. Qual..

[7] Reimann C (2008). Element levels in birch and spruce wood ashes—green energy?. Sci. Total Environ..

[8] Falkowski PG, Fenchel T, Delong EF (2008). The microbial engines that drive Earth's biogeochemical cycles. Science.

[9] Rønn R, Vestergard M, Ekelund F (2012). Interactions between bacteria, protozoa and nematodes in soil. Acta Protozool..

[10] van der Heijden MGA, Bardgett RD, van Straalen NM (2008). The unseen majority: soil microbes as drivers of plant diversity and productivity in terrestrial ecosystems. Ecol. Lett..

[11] Wall DH (2012). Soil Ecology and Ecosystem Services.

[12] Fierer N (2017). Embracing the unknown: disentangling the complexities of the soil microbiome. Nat. Rev. Microbiol..

[13] Kaiser K (2016). Driving forces of soil bacterial community structure, diversity, and function in temperate grasslands and forests. Sci. Rep..

[14] Waldrop MP, Balser TC, Firestone MK (2000). Linking microbial community composition to function in a tropical soil. Soil Biol. Biochem..

[15] Bang-Andreasen T (2017). Wood ash induced pH changes strongly affect soil bacterial numbers and community composition. Front. Microbiol..

[16] Bååth E, Arnebrant K (1994). Growth-rate and response of bacterial communities to pH in limed and ash treated forest soils. Soil. Biol. Biochem..

[17] Cruz-Paredes C, Wallander H, Kjøller R, Rousk J (2017). Using community trait-distributions to assign microbial responses to pH changes and Cd in forest soils treated with wood ash. Soil. Biol. Biochem..

[18] Fritze H, Perkiömäki J, Pennanen T (2000). Distribution of microbial biomass and phospholipid fatty acids in Podzol profiles under coniferous forest. Eur. J. Soil Sci..

[19] Frostegård A, Bååth E, Tunlid A (1993). Shifts in the structure of soil microbial communities in limed forests as revealed by phospholipid fatty-acid analysis. Soil. Biol. Biochem..

[20] Jokinen HK, Kiikkilä O, Fritze H (2006). Exploring the mechanisms behind elevated microbial activity after wood ash application. Soil. Biol. Biochem..

[21] Noyce GL (2016). Soil microbial responses to wood ash addition and forest fire in managed Ontario forests. Appl. Soil Ecol..

[22] Perkiömäki J, Fritze H (2002). Short and long-term effects of wood ash on the boreal forest humus microbial community. Soil. Biol. Biochem..

[23] Vestergård M (2018). The relative importance of the bacterial pathway and soil inorganic nitrogen increase across an extreme wood-ash application gradient. GBC Bioenergy.

[24] Fierer N, Jackson RB (2006). The diversity and biogeography of soil bacterial communities. Proc. Natl. Acad. Sci. USA.

[25] Rousk J (2010). Soil bacterial and fungal communities across a pH gradient in an arable soil. ISME J..

[26] Demeyer A, Nkana JCV, Verloo MG (2001). Characteristics of wood ash and influence on soil properties and nutrient uptake: an overview. Bioresour. Technol..

[27] Maresca A, Hyks J, Astrup TF (2017). Recirculation of biomass ashes onto forest soils: ash composition, mineralogy and leaching properties. Waste Manag..

[28] Fierer N, Bradford MA, Jackson RB (2007). Toward an ecological classification of soil bacteria. Ecology.

[29] Nemergut DR, Cleveland CC, Wieder WR, Washenberger CL, Townsend AR (2010). Plot-scale manipulations of organic matter inputs to soils correlate with shifts in microbial community composition in a lowland tropical rain forest. Soil. Biol. Biochem..

[30] Philippot L (2010). The ecological coherence of high bacterial taxonomic ranks. Nat. Rev. Microbiol..

[31] Ramirez KS, Craine JM, Fierer N (2012). Consistent effects of nitrogen amendments on soil microbial communities and processes across biomes. Glob. Change Biol..

[32] Gömöryová E, Pichler V, Tóthová S, Gömöry D (2016). Changes of chemical and biological properties of distinct forest floor layers after wood ash application in a Norway spruce stand. Forests.

[33] Hansen M, Bang-Andreasen T, Sørensen H, Ingerslev M (2017). Micro vertical changes in soil pH and base cations over time after application of wood ash on forest soil. For. Ecol. Manag..

[34] Blume E (2002). Surface and subsurface microbial biomass, community structure and metabolic activity as a function of soil depth and season. Appl. Soil. Ecol..

[35] Ekelund F, Rønn R, Christensen S (2001). Distribution with depth of protozoa, bacteria and fungi in soil profiles from three Danish forest sites. Soil Biol. Biochem..

[36] Fierer N, Schimel JP, Holden PA (2003). Variations in microbial community composition through two soil depth profiles. Soil Biol. Biochem..

[37] Drew MC (1975). Comparison of effects of a localized supply of phosphate, nitrate, ammonium and potassium on growth of seminal root system, and shoot, in Barley. New Phytol..

[38] Hutchings MJ, John EA (2004). The effects of environmental heterogeneity on root growth and root/shoot partitioning. Ann. Bot..

[39] Brunner I, Zimmermann S, Zingg A, Blaser P (2004). Wood-ash recycling affects forest soil and tree fine-root chemistry and reverses soil acidification. Plant Soil..

[40] Saarsalmi A, Smolander A, Moilanen M, Kukkola M (2014). Wood ash in boreal, low-productive pine stands on upland and peatland sites: long-term effects on stand growth and soil properties. For. Ecol. Manag..

[41] Lanzén A (2015). The community structures of prokaryotes and fungi in mountain pasture soils are highly correlated and primarily influenced by pH. Front. Microbiol..

[42] Lauber CL, Hamady M, Knight R, Fierer N (2009). Pyrosequencing-based assessment of soil pH as a predictor of soil bacterial community structure at the continental scale. Appl. Environ. Microbiol..

[43] Bang-Andreasen T, Schostag M, Prieme A, Elberling B, Jacobsen CS (2017). Potential microbial contamination during sampling of permafrost soil assessed by tracers. Sci. Rep..

[44] Saarsalmi A, Kukkola M, Moilanen M, Arola M (2006). Long-term effects of ash and N fertilization on stand growth, tree nutrient status and soil chemistry in a Scots pine stand. For. Ecol. Manag..

[45] Zimmermann S, Frey B (2002). Soil respiration and microbial properties in an acid forest soil: effects of wood ash. Soil Biol. Biochem..

[46] Bååth E (1996). Adaptation of soil bacterial communities to prevailing pH in different soils. Fems Microbiol. Ecol..

[47] Madigan MT, Martinko JM, Dunlap PV, Clark DP (2014). Brock Biology of Microorganisms.

[48] Rosso L, Lobry JR, Bajard S, Flandrois JP (1995). Convenient model to describe the combined effects of temperature and pH on microbial-growth. Appl. Environ. Microb..

[49] Kielak AM, Barreto CC, Kowalchuk GA, van Veen JA, Kuramae EE (2016). The ecology of acidobacteria: moving beyond genes and genomes. Front. Microbiol..

[50] Kim JM (2016). Soil pH and electrical conductivity are key edaphic factors shaping bacterial communities of greenhouse soils in Korea. J. Microbiol..

[51] Ochecova P, Tlustos P, Szakova J, Mercl F, Maciak M (2016). Changes in nutrient plant availability in loam and sandy clay loam soils after wood fly and bottom ash amendment. Agron. J..

[52] Pitman RM (2006). Wood ash use in forestry—a review of the environmental impacts. Forestry.

[53] Cederlund H (2014). Soil carbon quality and nitrogen fertilization structure bacterial communities with predictable responses of major bacterial phyla. Appl. Soil Ecol..

[54] Cleveland CC, Nemergut DR, Schmidt SK, Townsend AR (2007). Increases in soil respiration following labile carbon additions linked to rapid shifts in soil microbial community composition. Biogeochemistry.

[55] Padmanabhan P (2003). Respiration of C-13-labeled substrates added to soil in the field and subsequent 16S rRNA gene analysis of C-13-labeled soil DNA. Appl. Environ. Microbiol..

[56] Lladó S, Baldrian P (2017). Community-level physiological profiling analyses show potential to identify the copiotrophic bacteria present in soil environments. PLoS ONE.

[57] Starke R (2016). Bacteria dominate the short-term assimilation of plant-derived N in soil. Soil Biol. Biochem..

[58] Teng Y, Wang XM, Li LN, Li ZG, Luo YM (2015). Rhizobia and their bio-partners as novel drivers for functional remediation in contaminated soils. Front. Plant Sci..

[59] Bergmann GT (2011). The under-recognized dominance of Verrucomicrobia in soil bacterial communities. Soil Biol. Biochem..

[60] Hansen M, Saarsalmi A, Peltre C (2016). Changes in SOM composition and stability to microbial degradation over time in response to wood chip ash fertilisation. Soil Biol. Biochem..

[61] Reid C, Watmough SA (2014). Evaluating the effects of liming and wood-ash treatment on forest ecosystems through systematic meta-analysis. Can. J. For. Res..

[62] Levy-Booth DJ (2007). Cycling of extracellular DNA in the soil environment. Soil Biol. Biochem..

[63] Nielsen KM, Johnsen PJ, Bensasson D, Daffonchio D (2007). Release and persistence of extracellular DNA in the environment. Environ. Biosaf. Res..

[64] Carini P (2017). Relic DNA is abundant in soil and obscures estimates of soil microbial diversity. Nat. Microbiol..

[65] Carvalhais LC, Dennis PG, Tyson GW, Schenk PM (2012). Application of metatranscriptomics to soil environments. J. Microbiol. Methods.

[66] Urich T (2008). Simultaneous assessment of soil microbial community structure and function through analysis of the meta-transcriptome. PLoS ONE.

[67] Bang-Andreasen T (2019). Total RNA sequencing reveals multilevel microbial community changes and functional responses to wood ash application in agricultural and forest soil. FEMS Microbiol. Ecol..

[68] Zhang J, Kobert K, Flouri T, Stamatakis A (2014). PEAR: a fast and accurate Illumina Paired-End reAd mergeR. Bioinformatics.

