# Supplementary material for: Application of wood ash leads to strong vertical gradients in soil pH changing prokaryotic community structure in forest top soil
Source: Sci Rep. 2021 Jan 12;11:742. doi: 10.1038/s41598-020-80732-0 (PMC7804945; doi:10.1038/s41598-020-80732-0)
Supplement: Supplementary file 1 — Supplementary Information. [file 41598_2020_80732_MOESM1_ESM.docx]

**Supplementary information**

**Application of wood ash leads to strong vertical gradients in soil pH changing prokaryotic community structure in forest top soil**

Toke Bang-Andreasen^1,2^, Mette Peltre^3^, Lea Ellegaard-Jensen^1^, Lars Hestbjerg Hansen^1,4^, Morten Ingerslev^3^, Regin Rønn^2^, Carsten Suhr Jacobsen^1^, Rasmus Kjøller^2^

^1^Department of Environmental Science, Aarhus University, Roskilde, Denmark. ^2^Department of Biology, University of Copenhagen, Copenhagen, Denmark. ^3^Department of Geosciences and Natural Resource Management, University of Copenhagen, Denmark. ^4^Department of Plant and Environmental Sciences, University of Copenhagen.

Corresponding author: Rasmus Kjøller, rasmusk@bio.ku.dk, +45 41432331, 0000-0002-2027-4119


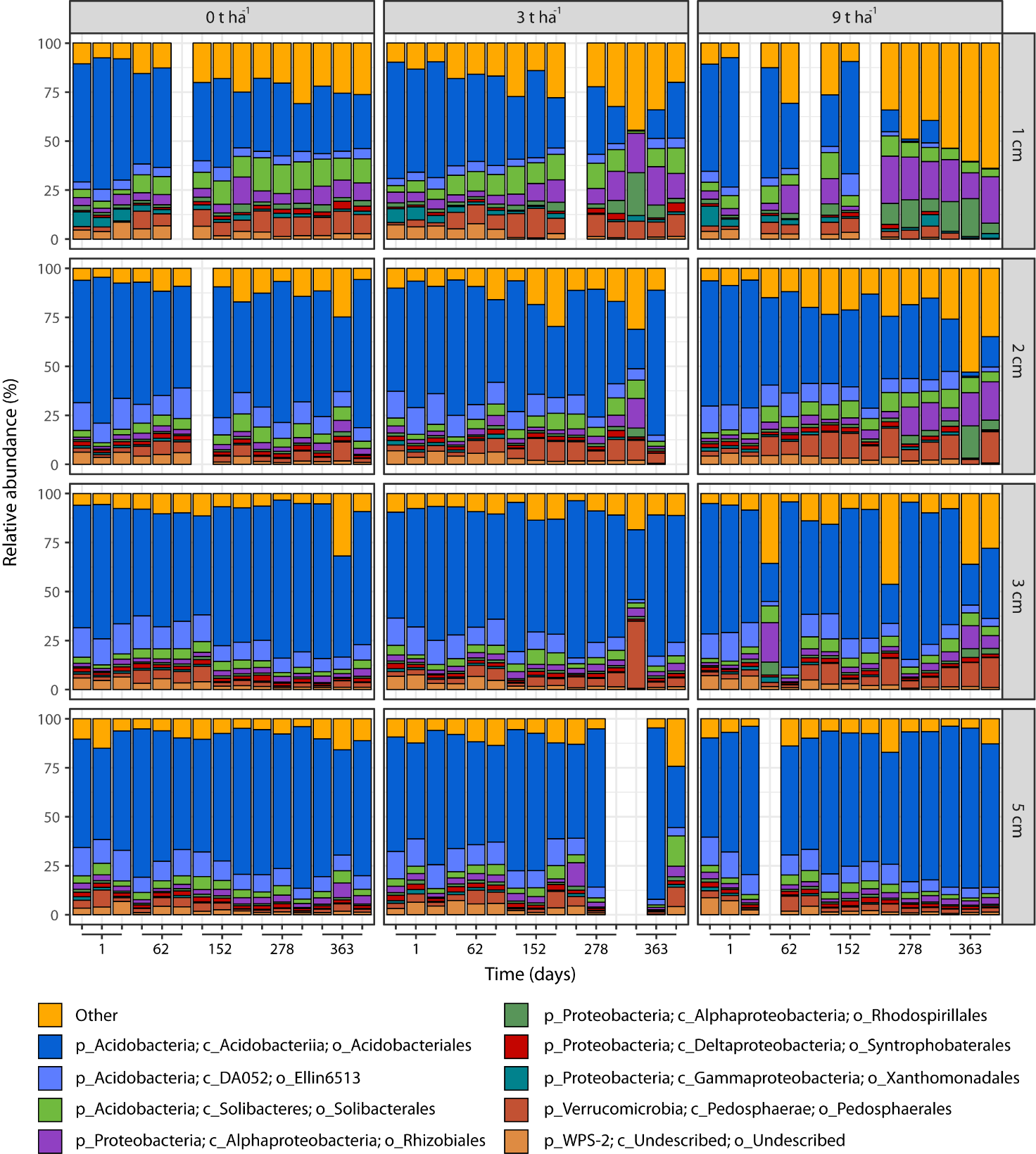


**Supplementary Figure 1.** Most dominant prokaryotic orders (>1% average relative abundance) across wood ash concentration, soil depth and time after wood ash application. Each replicate within each time point and ash concentration are presented. Empty bar plots represents samples removed because of too few DNA sequences were obtained (threshold of 5000 sequences per sample). “p_”, “c_” and “o_” denote taxonomic rank of phylum, class and order, respectively. Figure was created in R v. 1.0.153 (www.R-project.org) and GIMP v. 2.8.22 (https://www.gimp.org)


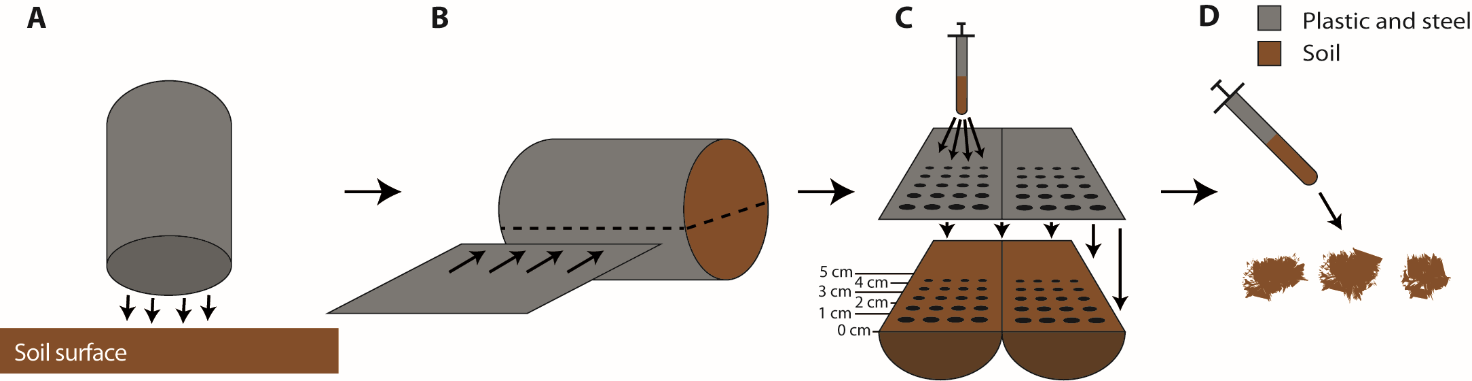


**Supplementary Figure 2.** Soil collection procedure to avoid microbial contamination dragged from upper soil layers during collection of soil cores: (A) Soil cores collected by pushing plastic tubes (Ø = 6 cm) into the soil. (B) Inside a laminar air flow bench using sterile tools, plastic tubes were slightly opened longitudinal in one side allowing a stainless steel plate to be pushed through the soil core from one side to the other splitting the core into two longitudinal half’s. (C) The two half’s were then separated and collection of soil was done from these newly exposed surfaces using a plastic template which allowed 1 ml syringes (BD Tuberculin Syringe 1 ml) with the tip removed to collect soil from fixed 1 cm depth intervals. Syringes were not pushed closer than 1 cm to the rounded surface of the cores thereby avoiding the potential microbial contamination present in the outermost layers of the cores. (D) Uncontaminated soil retrieved. This procedure is based on a general procedure to sample soil cores with special emphasis on avoiding contamination (Bang-Andreasen et al., 2017)

**Supplementary Table 1:** Cation exchange capacity (CEC) and NH_4_NO_3_ extractable cations Ca, K, Mg, Mn, Na, Fe and Al across wood ash across wood ash applications (0, 3 and 9 t ha^-1^), time after ash application and soil depth. Bold values represents significantly different values from unamended (0 t ha^-1^) soil samples. Italic values indicate significantly different values between samples with ash doses 3 and 9 t ha^-1^. Data From Hansen et al. (2017).

|  |  |  | 0 t ha^-1^ | | | |  | 3 t ha^-1^ | | | |  | 9 t ha^-1^ | | | |
| --- | --- | --- | --- | --- | --- | --- | --- | --- | --- | --- | --- | --- | --- | --- | --- | --- |
|  | Depth |  | 62 d | 152 d | 278 d | 363 d |  | 62 d | 152 d | 278 d | 363 d |  | 62 d | 152 d | 278 d | 363 d |
|  |  |  |  |  |  |  |  |  |  |  |  |  |  |  |  |  |
| CEC  cmol(+) kg^-1^ | 0-1 cm |  | 18.8 | 19.6 | 17.3 | 19.5 |  | **41.1** | **41.1** | **30.1** | **17.1** |  | **46.3** | **44.3** | **37.9** | **27.6** |
|  | 1-2 cm |  | 5.87 | 5.41 | 4.69 | 4.07 |  | 5.81 | 5.20 | 11.9 | 5.32 |  | ***3.99*** | ***7.00*** | ***19.6*** | ***20.1*** |
|  | 2-3 cm |  | 4.02 | 4.11 | 3.29 | 3.32 |  | 3.55 | 2.97 | 3.79 | 3.67 |  | 3.21 | 4.05 | 4.16 | 10.7 |
|  | 4-5 cm |  | 3.88 |  |  | 3.60 |  | 3.15 |  |  | 3.87 |  | 3.51 |  |  | 4.01 |
|  |  |  |  |  |  |  |  |  |  |  |  |  |  |  |  |  |
| Ca  mg kg^-1^ | 0-1 cm |  | 237 | 235 | 177 | 204 |  | **3276** | **3293** | **3001** | **1756** |  | **3732** | **3313** | **3551** | **2863** |
|  | 1-2 cm |  | 36.9 | 38.4 | 83.1 | 135 |  | 286 | 235 | 1879 | 717 |  | **189** | **380** | **3127** | **3234** |
|  | 2-3 cm |  | 15.5 | 17.2 | 51.1 | 49.5 |  | 52.7 | 57.6 | 357 | 320 |  | **42.0** | **163** | **490** | **1111** |
|  | 4-5 cm |  | 10.9 |  |  | 52.1 |  | 11.0 |  |  | 72.4 |  | ***19.4*** |  |  | ***115*** |
|  |  |  |  |  |  |  |  |  |  |  |  |  |  |  |  |  |
| K  mg kg^-1^ | 0-1 cm |  | 358 | 309 | 264 | 274 |  | **2603** | **1794** | **765** | **455** |  | **2774** | **2493** | **1277** | **697** |
|  | 1-2 cm |  | 74.0 | 64.8 | 65.9 | 72.7 |  | 472 | 290 | 156 | 43.1 |  | **375** | **579** | **331** | **245** |
|  | 2-3 cm |  | 36.8 | 35.9 | 25.4 | 25.7 |  | 163 | 136 | 78.8 | 68.2 |  | **161** | **318** | **106** | **138** |
|  | 4-5 cm |  | 28.9 |  |  | 17.5 |  | 56.0 |  |  | 79.5 |  | **934** |  |  | **147** |
|  |  |  |  |  |  |  |  |  |  |  |  |  |  |  |  |  |
| Mg  mg kg^-1^ | 0-1 cm |  | 449 | 480 | 416 | 469 |  | **1834** | **2090** | **1356** | **723** |  | **2150** | **2221** | **1806** | **1216** |
|  | 1-2 cm |  | 84.8 | 82.6 | 55.1 | 78.5 |  | 217 | 220 | 180 | 91.0 |  | **143** | **304** | **333** | **363** |
|  | 2-3 cm |  | 38.1 | 42.3 | 26.7 | 33.4 |  | 56.4 | 65.5 | 68.7 | 65.6 |  | **45.8** | **116** | **96.8** | **216** |
|  | 4-5 cm |  | 29.3 |  |  | 33.7 |  | 23.3 |  |  | 63.7 |  | **32.4** |  |  | **65.8** |
|  |  |  |  |  |  |  |  |  |  |  |  |  |  |  |  |  |
| Mn  mg kg^-1^ | 0-1 cm |  | 11.1 | 12.5 | 8.50 | 10.2 |  | **248** | **268** | **353** | **218** |  | **233** | **192** | **237** | **225** |
|  | 1-2 cm |  | 2.09 | 2.12 | 1.69 | 1.95 |  | 42.5 | 34.6 | 44.0 | 15.4 |  | **36.5** | **57.5** | **49.1** | **53.5** |
|  | 2-3 cm |  | 0.988 | 1.15 | 1.12 | 1.02 |  | 10.1 | 11.1 | 8.04 | 6.52 |  | **8.69** | **27.6** | **11.5** | **30.1** |
|  | 4-5 cm |  | 0.755 | |  | 1.18 |  | 1.42 |  |  | 2.04 |  | **3.42** |  |  | **4.78** |
|  |  |  |  |  |  |  |  |  |  |  |  |  |  |  |  |  |
| Na  mg kg^-1^ | 0-1 cm |  | 185 | 130 | 112 | 119 |  | **466** | **420** | **153** | **93.9** |  | **458** | **536** | **275** | **152** |
|  | 1-2 cm |  | 37.9 | 238 | 23.2 | 24.3 |  | 95.0 | 54.5 | 29.9 | 15.1 |  | 57.6 | 100 | 58.3 | 35.2 |
|  | 2-3 cm |  | 19.2 | 14.7 | 12.9 | 12.4 |  | 32.7 | 26.0 | 13.5 | 12.6 |  | **26.2** | **53.9** | **19.3** | **25.4** |
|  | 4-5 cm |  | 14.4 |  |  | 11.1 |  | 15.7 |  |  | 16.2 |  | **18.3** |  |  | **24.6** |
|  |  |  |  |  |  |  |  |  |  |  |  |  |  |  |  |  |
| Fe  mg kg^-1^ | 0-1 cm |  | 71.3 | 85.1 | 78.2 | 140 |  | **9.10** | **9.96** | **7.23** | **6.04** |  | **9.26** | **9.14** | **7.65** | **5.75** |
|  | 1-2 cm |  | 21.5 | 21.8 | 27.1 | 37.0 |  | **3.56** | **6.22** | **1.89** | **5.42** |  | **3.57** | **2.77** | **1.93** | **1.73** |
|  | 2-3 cm |  | 13.2 | 18.0 | 15.8 | 20.7 |  | **11.0** | **10.5** | **9.34** | **10.2** |  | ***9.86*** | ***6.36*** | ***3.97*** | ***2.01*** |
|  | 4-5 cm |  | 13.1 |  |  | 27.4 |  | 10.7 |  |  | 24.6 |  | **11.5** |  |  | **25.4** |
|  |  |  |  |  |  |  |  |  |  |  |  |  |  |  |  |  |
| Al  mg kg^-1^ | 0-1 cm |  | 163 | 176 | 172 | 214 |  | **2.43** | **1.79** | **2.29** | **2.91** |  | **0.908** | **1.58** | **0.664** | **0.406** |
|  | 1-2 cm |  | 80.8 | 60.9 | 61.4 | 66.5 |  | **7.15** | **12.7** | **2.66** | **8.59** |  | **7.59** | **4.42** | **0.840** | **0.644** |
|  | 2-3 cm |  | 61.3 | 58.8 | 49.1 | 49.7 |  | **41.0** | **27.2** | **21.5** | **22.0** |  | **37.8** | **17.1** | **7.02** | **2.32** |
|  | 4-5 cm |  | 63.1 |  |  | 57.9 |  | **47.7** |  |  | **53.9** |  | ***52.9*** |  |  | ***48.2*** |

**Supplementary Table 2:** R2-values from *Adonis* (PERMANOVA) testing. (A) Wood ash concentration (Conc), time after wood ash application (Time) and the interaction of these as explanatory variables for the observed community dissimilarities between samples (Bray-Curtis) within each soil depth. (B) The same test as in (A) but seperately on samples within each combination of soil depth and time point. This results in testing of wood ash concentration as explanatory variable within each time point and soil depth. Asterisk represents significance level (* is 0.01<p<0.05, ** is 0.001<p<0.05, *** is p< 0.001).

| **A** |  |  |  |  |  |
| --- | --- | --- | --- | --- | --- |
| Depth | Conc | Time | Conc:Time |  |  |
| 0.5 cm | 0.206*** | 0.253*** | 0.173 |  |  |
| 1.5 cm | 0.084** | 0.226*** | 0.210* |  |  |
| 2.5 cm | 0.086** | 0.217*** | 0.157 |  |  |
| 4.5 cm | 0.081* | 0.159** | 0.208 |  |  |
|  |  |  |  |  |  |
| **B** |  |  |  |  |  |
| Depth | 1 d | 62 d | 152 d | 278 d | 363 d |
| 0.5 cm | 0.285 | 0.579* | 0.371 | 0.539* | 0.552** |
| 1.5 cm | 0.423 | 0.307 | 0.401 | 0.376 | 0.392 |
| 2.5 cm | 0.209 | 0.277 | 0.398 | 0.295 | 0.319 |
| 4.5 cm | 0.234 | 0.438 | 0.364 | 0.294 | 0.348 |

**Supplementary References**

Bang-Andreasen, T., Schostag, M., Prieme, A., Elberling, B. & Jacobsen, C. S., 2017. Potential microbial contamination during sampling of permafrost soil assessed by tracers. Scientific Reports 43338 (2017)

Hansen, M., Bang-Andreasen, T., Sørensen, H., Ingerslev, M., 2017. Micro vertical changes in soil pH and base cations over time after application of wood ash on forest soil. Forest Ecology and Management 406, 274–280. doi:10.1016/j.foreco.2017.09.069
